# Effectiveness evaluation of autotransplanted teeth after performing extraoral endodontic surgery instead of conventional root canal therapy

**DOI:** 10.1186/s12903-023-03733-1

**Published:** 2023-12-14

**Authors:** Fenglin Liao, Hui Wang, Jihong Zhao, Biao Zhang, Haoyan Zhong

**Affiliations:** 1 State Key Laboratory of Oral & Maxillofacial Reconstruction and Regeneration, Key Laboratory of Oral Biomedicine Ministry of Education, Hubei Key Laboratory of Stomatology, School & Hospital of Stomatology, Wuhan University, Wuhan, China; 2 Department of Oral and Maxillofacial Surgery, School and Hospital of Stomatology, Wuhan University, Wuhan, China; 3 Department of Endodontics,School and Hospital of Stomatology, Wuhan University, Wuhan, China

**Keywords:** Autotransplanted teeth, Apicoectomy, Chewing efficiency, Retrograde filling, Retropreparation, Survival and success rate

## Abstract

**Purpose:**

The aim of this study was to examine the viability and efficacy of utilizing extraoral apicoectomy and retrograde filling in combination to seal the root canal system of mature molars without the need for root canal therapy (RCT) during the autotransplantation of teeth (ATT).

**Materials and methods:**

This study screened 27 patients who received ATT at the Department of Oral Surgery in the Hospital of Stomatology from 2019 to 2021. Extraoral apicoectomy and retrograde filling were performed, while RCT was temporarily not performed. The study analysed the periodontal status and masticatory function of transplanted teeth one to three years postoperation and used cone-beam computed tomography (CBCT) and periapical radiograph (PA) to evaluate the integrity of the periodontal space and intra/periapical inflammation. The potential predictors of survival/success were analysed statistically. We also conducted questionnaires and chewing efficiency tests.

**Results:**

In this study, 27 TTs from 27 patients were found to be fully functional in terms of chewing ability. The overall survival rate was 100% (27/27), and the success rate was 70.4% (19/27). A total of 90.9% (20/22) of patients reported being satisfied or very satisfied with their TTs. Additionally, the chewing efficiency of the transplantation side was on average 82.0% of that of the healthy side, with a significant difference between the two sides (*P* < 0.05). None of the potential predictors were found to significantly affect the success or survival of the transplanted tooth (TT).

**Conclusion:**

The combination of extraoral apicoectomy and retrograde filling in TT showed promising outcomes, but further clinical cases and longer follow-up times are still required to validate the treatment plan.

## Introduction

Autotransplantation of tooth (ATT) is a surgical procedure that involves transplanting an individual’s own tooth from one location to another [1–3]. This method is commonly used to move nonchewing teeth to other extraction sockets or surgically prepared alveolar fossae [4]. ATT is a biocompatible rehabilitation technique that effectively repairs dental defects and preserves alveolar bone mass, allowing the natural tooth to be utilized to its fullest extent [1, 3, 5]. With the further development of oral instruments and related technologies, the success rate of ATT has improved in clinical research [4, 6, 7]. Compared to ATT, intentional replantation is defined as the deliberate extraction of a tooth and after evaluating the root surfaces, performing endodontic manipulation and repair, the tooth is then placed back into its original position [7]. This economical and practical technique has been widely praised by dentists and patients alike for its practicality and affordability [3, 5].

Traditionally, it has been recommended to perform root canal therapy (RCT) on mature donor teeth within 2 weeks after surgery [6, 8], with crown restoration suggested at a later stage. However, the intricate and varied nature of root canal systems in wisdom teeth presents considerable difficulties when performing RCTs, despite advancements in technology [9–11]. Failure to fully complete RCT can ultimately lead to failure of the ATT [6, 8].

Furthermore, the cost of ATT treatment has significantly increased due to the need for RCT and crown restoration [12, 13]. If the extraoral apicoectomy and retrograde filling method can be performed without postoperative RCT, it has the potential to significantly reduce patient time and economic costs, as well as reduce the risks and difficulties associated with ATT [14].

According to clinical studies, patients who opt for apicoectomy or apicoectomy with retro-filling instead of conventional RCT for ATT may experience favorable clinical outcomes. This claim is backed by published case reports and research [15–20].

This study aimed to observe the status and repair effect of transplanted teeth (TTs) in patients who underwent extraoral apicoectomy and retrograde filling without RCT. The primary objective was to evaluate the survival rate and success rate of the TT, while secondary objectives included investigating patient satisfaction with the TT, recovery of chewing efficiency and factors affecting the success rate, such as fused root and separate roots, as well as recording relevant clinical and radiographic parameters. The follow-up period for the study was at least 17 months. The findings of this study can provide new insights and serve as a reference for ATT techniques.

## Material and method

### Patient selection

The study included 27 autotransplantation cases of mature permanent teeth during donor extraction who were treated between July 2019 and June 2021 at the Department of Oral Surgery in the Hospital of Stomatology. All patients underwent extraoral apicoectomy and retrograde filling using iRoot BP plus (Innovative Bioceramix, Inc.; British Columbia Canada V3N 0E9) during the ATT, without conventional RCT temporarily. Cases were recorded at least 17 months after ATT, and patients with decent compliance and signed informed consent were included in the study. Those who were unable or unwilling to cooperate for subjective or objective reasons were excluded from the observational study. The study protocol was approved by the standing ethics review board (Ethics Committee of Hospital of Stomatology, approval number: [2021]伦审字 (B58), and the investigation was performed in accordance with the Declaration of Helsinki (2013).

### Surgical procedure

**Fig. 1:**
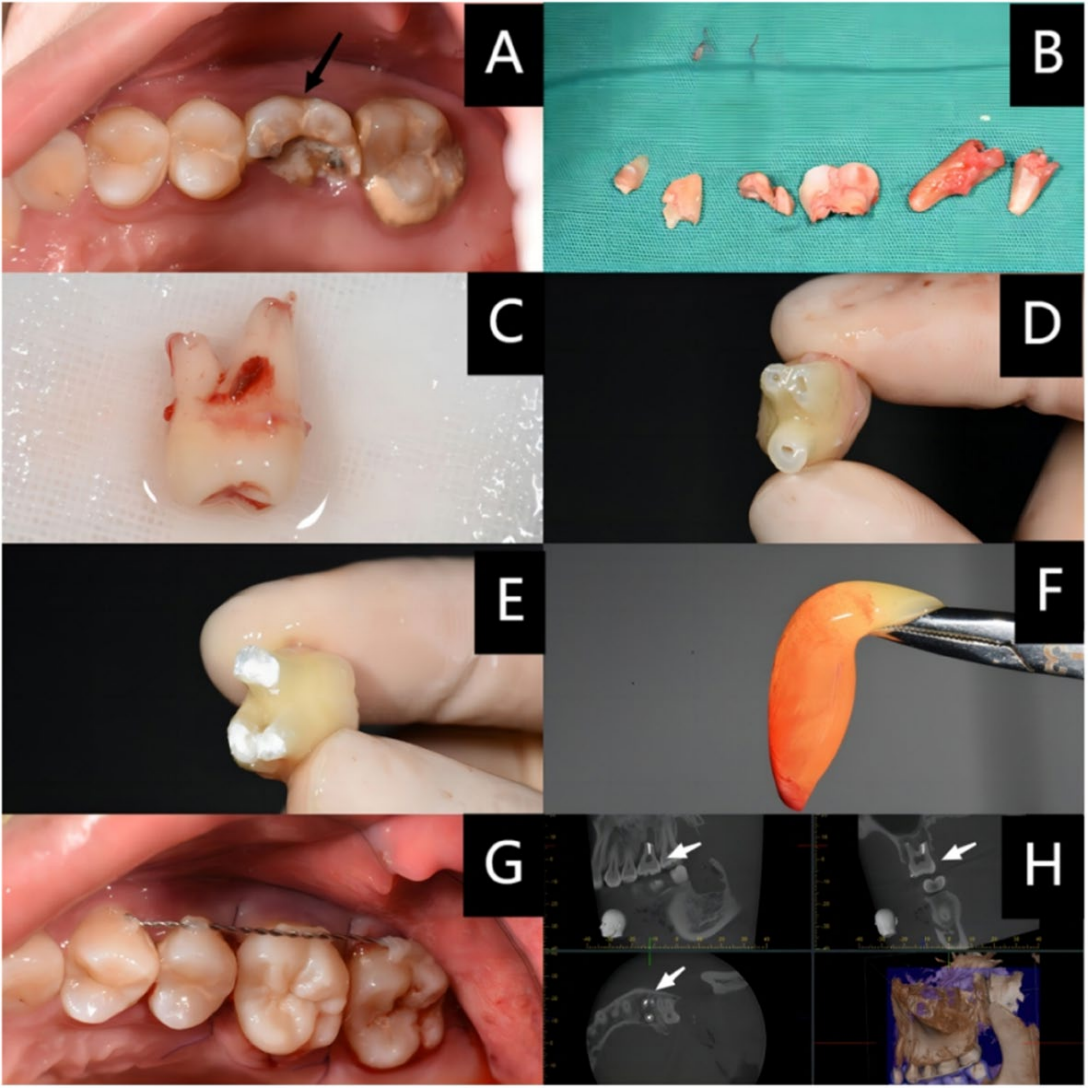
Residual crown in maxillary posterior region (**A**); Extraction of residual crown (**B**); Donor tooth with partially broken root (**C**); Apical trimming and retrograde preparation (**D**); IRoot BP plus filling (**E**); CGF for placement of alveolar socket to promote healing (**F**); Fixation with elastic ligature wire and flowable resin (**G**); CBCT scan was taken immediately after the procedure (**H**)

The patient received both block anaesthesia of the alveolar nerve using 2% lidocaine and infiltration anaesthesia using 4% articaine and epinephrine (1:100,000) prior to the extraction of the affected tooth. Standardized surgical instruments and minimally invasive techniques were utilized during the procedure. During the extraction process, it was essential to handle the donor tooth with care to minimize trauma. Once the tooth had been loosened, dental forceps were used to gently remove it. The donor tooth was then immediately placed in a container of normal saline at four degrees Celsius for storage. Before preparing the tooth root, the crown was wrapped in saline gauze. When holding the tooth, it was vital to do so lightly to avoid damaging the periodontal ligament (PDL). In this study, the root apex was trimmed by approximately 3 mm using a sterile bur and then rinsed with normal saline. If the apex of the donor tooth was broken during extraction and the breakage was less than 1/3 of the root length, then only the wound surface needed to be trimmed without performing an apicoectomy.) (Fig. 1 C/D). A piezosurgery tool with a work tip (UE1, Woodpecker Medical in Guilin, China) was used to retro-operate the root to a depth of 3 mm. Retrograde filling using iRoot BP plus was performed, with normal saline used to keep the root surface moist throughout the process. Following the completion of root preparation, the donor tooth was transplanted into the alveolar socket of the recipient area to assess the degree of matching. Subsequently, the crown was gently removed using minimally invasive dental forceps and placed in a solution of normal saline. The alveolar socket was then prepared using a sterilized dental electric handpiece, with cooling provided by a saline solution throughout. During the process of donor tooth transplantation, the implantation position could be adjusted appropriately to obtain an optimal functional position for the donor tooth, and the enamelo-cemental junction was placed at 2–3 mm below the gingival margin. Once the donor tooth was in place, it was secured using elastic ligature wires and flow resin. As needed, the occlusion was lifted using glass ions. The short tuning procedure is shown in Fig. 1 below. The patient was required to eat on the healthy side for at least 4 weeks, and the fixation device was removed at a follow-up visit 4 weeks later. Consistency was maintained in the materials and equipment used for all patients.

### Data collection

1. Telephone follow-up

Telephone follow-up was conducted for all patients after surgery to determine the survival of the TT. Additionally, clinical and radiographic examinations were arranged and performed.

(2) Clinical evaluation

Follow-up patients underwent clinical and radiographic evaluations conducted by a clinician who was not involved in the surgical procedure. The clinical assessment included an evaluation of percussion response, degree of mobility, gingival index, periodontal depth, TMD status, Ohi-s index, bruxism, occlusion relationship, and other relevant factors.

(3) Radiographic evaluation

The evaluation items include the integrity of the periodontal space and the intra/periapical radiolucencies. The PA (eXpert DC Pennsylvania USA) was taken, and images were assessed using Digora (Soredex, Helsinki, Finland). CBCT was taken by the same machine (Newtom VGI, Quantitative Radiology, Verona, Italy) in all included cases. The CBCT parameters were consistent. The image output format was DICOM 3.0 with a resolution of 0.30 mm for CT. The image analysis and processing software was New-TomNNT.

(4) Questionnaire survey

The questionnaire primarily focused on assessing the usage of the TT, the presence of any discomfort symptoms, and the patients’ satisfaction levels with their TTs.

(5) Chewing efficiency test

To compare the chewing efficiency between the transplantation and healthy side, we designed a test that involved measuring absorbance using an ultraviolet spectrophotometer (UV − 2700i model) at a wavelength range of 185–900 nm. The manufacturer of the equipment used was SHIMADZU Instrument (Suzhou) Co., Ltd., and the equipment origin was 183 Taishan Road, High-tech Zone, Suzhou City, Jiangsu Province, China.

Spectrophotometers measured the selective absorption of light by substances at specific wavelengths. Each substance had its own absorption bands, causing certain wavelengths of light to be absorbed as it passes through a solution. The degree of attenuation of light energy at a particular wavelength was proportional to the concentration of the substance in the solution [21]. According to the principle, the larger the surface area of food particles is, the less soluble substances there are, the smaller the solution concentration is, and the smaller the absorbance value is, resulting in lower chewing efficiency [22]. Conversely, higher chewing efficiency leads to a higher absorbance value. The ratio of chewing efficiency could be determined by comparing the absorbance value between the transplanted and healthy tooth sides, as shown in Table 3.

Thirteen patients who had previously received ATT agreed to participate in a chewing efficiency test experiment conducted 2 years later.

Peanuts were selected as the object for a chewing test. Before chewing, the subjects gargled with pure water. Weighed peanuts (1.00 ± 0.10 g) were then placed into the mouth, and the patients were instructed to chew on the transplantation side at a normal speed 10 times. After chewing, the material was transferred to a wide-mouth bottle and thoroughly mixed, and 50 mL of suspension was centrifuged. The absorbance value was measured at 590 nm using a spectrometer. The data were obtained by performing triplicate measurements in parallel and calculating the mean value. The same procedure was carried out on the healthy side. As the number of chews increased, the food particles decreased, and more soluble substances were released. This results in an increase in the concentration of the solution and ultimately leads to an increase in the absorbance value.

### Criteria

In the initial phase of the analysis, teeth that were extracted were considered failures. For the tooth examined during the follow-up, if the tooth was in place, without obvious pain and a tooth mobility score ≤ 2 (1 = slight mobility in the buccolingual direction, 2 = mobility in the buccolingual and mesiodistal directions, 3 = buccolingual, mesiodistal and vertical mobility) [23], they were considered alive, but success could not be determined. The term’success’ was defined based on the following criteria, which were adapted and modified from [24]: clinical criteria included the absence of pain, tooth mobility score (0–1), probing depth less than 3.5 mm, no signs of inflammation, and painless percussion. The radiographic criteria included a complete periodontal space and no signs of periapical or intraapical radiolucency.

During follow-up, donor teeth exhibiting one or more clinical or radiographic abnormalities were classified as potentially adverse findings and were therefore labelled ‘survival’.

### Statistical analysis

The chewing efficiency results were statistically analysed using GraphPad Prism 9.3.0 (GraphPad Prism) software. The distribution (normal or nonnormal) of the two groups of data was tested, and significant differences in the data were determined using paired t tests.

The satisfaction questionnaire results were computed, and relevant figures were drawn using Excel (Microsoft Excel 2019).

A log-rank test was used to compare the group differences between the fused root and the separate root. The survival probability was estimated using the Kaplan-Meier method, which refers to the success rate of donor teeth rather than the survival rate. The Cox proportional hazard model was used to analyse the potential factors affecting the success rates. The relevant statistical analysis was performed using SPSS software (SPSS/25.0, SPSS, Chicago, IL, USA).

*P* < 0.05 indicated a statistically significant difference.

## Results

### Basic information of patients

In total, 10 males and 17 females were included. Only one ATT surgery was performed on each patient. The mean age was 28.78 years. The oldest patient was 51 years old, and the youngest patient was 18 years old. The clinical features are summarized in Table 1. All donor teeth were completely developed permanent teeth with closed apical foramen. In total, 27 cases involved apicoectomy or trimming and retrograde filling. The surgery was performed by a trained and board-certified senior surgeon.

**Table 1:** Basic information of all patients

Patient			Perioperative		Follow up				
Transpla-nt,n	Gende-r	Age at surgery,y	Donor site	Recipi-ent site	Follow up,m	Follow up before symptom onset, m	RCT, y/n	Maximum depth of periodontal pocket examination, mm	Tooth mobilit-y
1	m	51	18	47	30	n	n	3	0
2	f	27	48	17	30	15	y	3	0
3	m	24	38	37	33	n	n	3	0
4	f	20	38	36	28	n	n	3	0
5	f	25	38	36	34	13	n	3	0
6	f	38	38	36	34	n	n	3	0
7	f	27	38	37	28	13	y	3	I°
8	f	28	28	46	36	36	n	2	0
9	f	31	48	47	35	n	n	3	0
10	m	24	28	27	35	n	n	3	0
11	m	29	48	46	41	41	y	3	0
12	m	34	48	47	37	n	n	3	0
13	f	28	48	36	31	n	n	2	0
14	m	48	48	46	29	n	n	3	0
15	f	39	38	36	30	n	n	3	0
16	f	26	28	36	33	n	y	3	0
17	m	23	48	46	36	n	n	3	0
18	f	26	48	47	29	n	n	3	0
19	f	23	48	46	29	n	n	2	0
20	f	32	28	27	29	n	n	2	0
21	m	23	37	37	29	n	n	3	0
22	f	22	28	26	24	n	n	3	0
23	f	32	48	46	19	n	n	2	0
24	f	23	38	37	23	23	y	3	0
25	m	18	48	46	23	20	y	3	0
26	m	32	38	37	21	18	y	5	0
27	f	24	38	36	17	n	n	4	0
Patient	Follow up								
Transplant,n	gingival index(max)		Percussio-n	TMD, y/n	OHI-S	bruxism,y/n	occlusion relation, R/L	Periradicu-lar radiolucen-cy,y/n	Periodon-tal space,y/n
1	0		–	n	6	y	I/I	n	y
2	2		±	n	6	n	I/I	y	y
3	2		±	n	4	n	III/III	n	y
4	0		–	n	1	n	I/I	n	y
5	2		±	n	6	n	I/II	n	y
6	0		–	n	3	n	I/I	n	y
7	2		–	n	6	n	I/III	y	y
8	0		–	y	1	n	II/II	n	y
9	0		–	n	3	n	I/I	n	y
10	0		–	n	3	n	I/I	n	y
11	0		–	n	6	n	I/I	y	y
12	0		–	n	13	n	II/II	n	y
13	2		–	n	7	n	I/I	n	y
14	2		–	n	10	n	I/II	n	y
15	2		–	n	1	n	III/I	n	y
16	2		±	n	3	y	I/I	y	y
17	0		–	n	6	n	I/I	n	y
18	0		–	n	6	n	I/I	n	y
19	0		–	n	6	n	I/I	n	y
20	1		–	n	4	n	I/I	n	y
21	2		–	n	6	n	I/I	n	y
22	2		–	y	6	n	I/I	n	y
23	2		–	n	3	y	I/I	n	y
24	2		–	y	1	n	I/III	y	y
25	0		–	n	2	n	III/III	y	y
26	3		–	n	6	y	I/I	y	y
27	2		–	n	1	y	II/II	n	y

(Follow-up data are available only for patients attending the examination. Timeframes are given in (m = months) and (y = years). Application of follow-up before symptom onset, TMD, RCT, bruxism, periradicular radiolucency or periodontal space are listed as (y = yes, n = no). Application of transplant is (n = number), and the gender framework is given in (m = male f = female)

The mean follow-up time for the 27 patients was 29.74 months, the survival rate of donor teeth was 100%, and the success rate was 70.4%. Among all 27 cases that were followed up, 8 cases eventually underwent RCT, but no one underwent crown restoration. No dental fracture or obvious cleft was found, and no crown discolouration was observed in any case. Eight of the 27 patients experienced adverse clinical or radiographic manifestations of TTs. The CBCT results from 8 cases showed varying levels of bone density loss in the apical region of the transplants, broadening of the periodontal space, and root resorption.

**Fig. 2:**
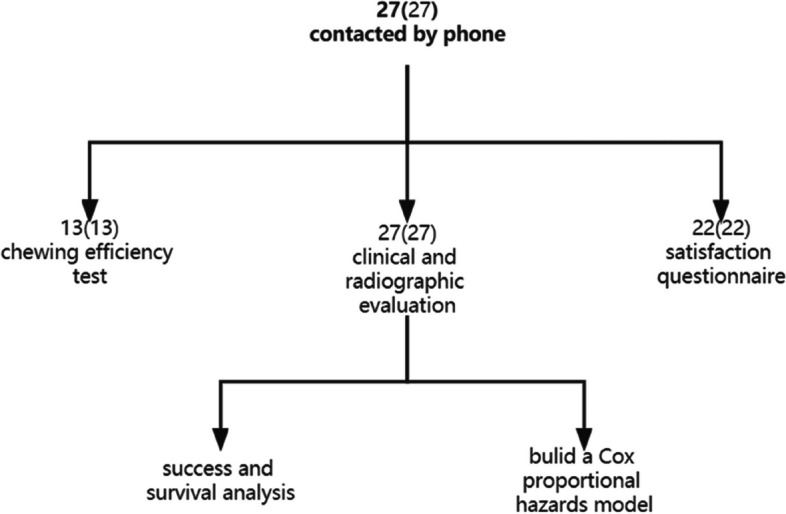
Flowchart for the analysis of autotransplanted teeth (patients)

**Fig. 3:**
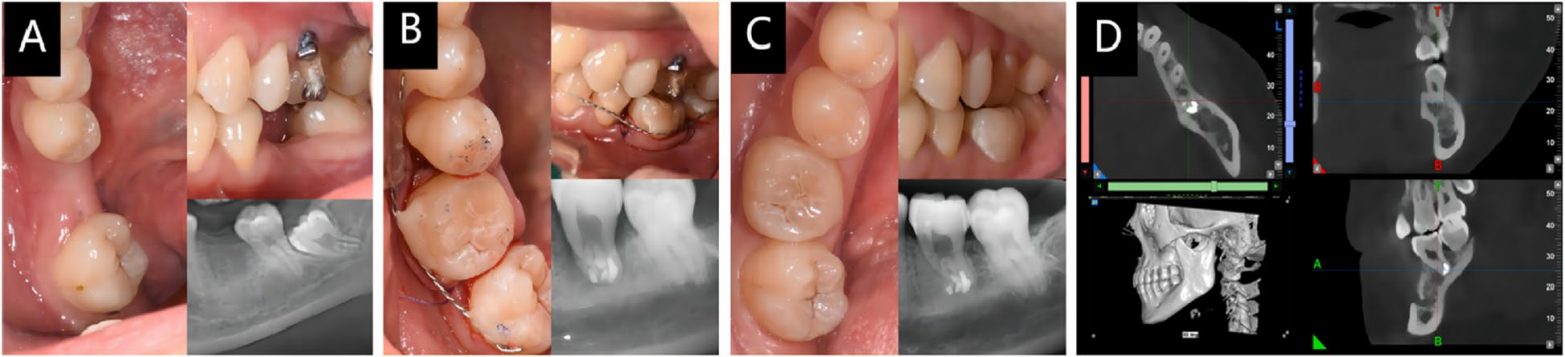
Preoperative clinical photographs and PA of the recipient area (**A**); clinical photos and PA of the recipient area immediately after surgery (**B**); clinical photos, PA and CBCT of the recipient area at 28 months after surgery (**C**, **D**)

Twenty-seven patients were contacted via telephone and agreed to undergo clinical and imaging examinations. A survival and success analysis was conducted, and a Cox proportional hazards model was developed. Of the 27 patients, 13 consented to a chewing efficiency test, and 22 patients completed a satisfaction survey about their TTs (Fig. 2). Figure 3 shows representative clinical and radiographic images of preoperative, intraoperative, and postoperative follow-up.

### Survival analysis

Nineteen transplants in 27 patients were asymptomatic, and CBCT showed that 8 cases showed intra- or peri-apical radiolucency approximately 17 months after transplantation, but there was no obvious discomfort in clinical examination, percussion (±), mobility (0 ~ 1), or the maximum depth of periodontal pocket examination was 5 mm. Therefore, the TT was not extracted, but the patients were still required to review regularly. After an average follow-up of 29.74 months, the survival rate of TT was 100% based on the survival criteria. Details are shown in Table 1.

### Success analysis

In this study, 27 TTs were categorized into three groups: fused root group (12 teeth), separate roots group (14 teeth), and single root group (1 tooth). The classification of fused and separated roots was based on the criteria defined in [25]. We used Kaplan-Meier analysis to estimate the 3-year success rate of the fused and separate roots group, which refers to the probability of having no adverse clinical and academic performance imaging results within 3 years. We used the log-rank test to compare the significant difference between the success rates of the fused and separate roots groups.

**Fig. 4:**
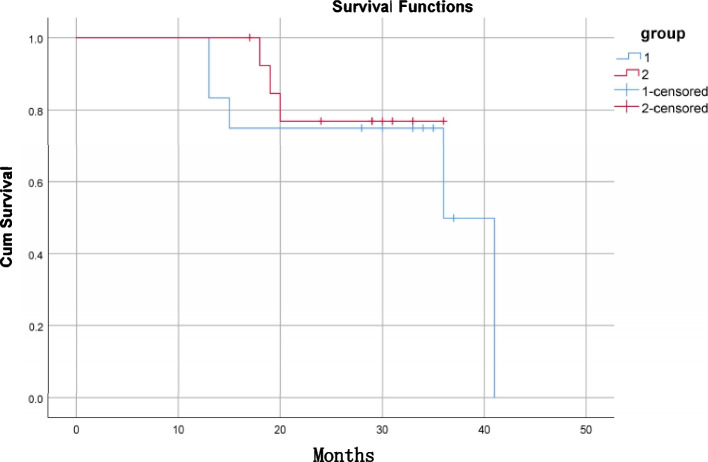
Kaplan–Meier survival probability of TTs; 1 = fused root group, 2 = separate roots group

The results showed that the Kaplan-Meier 3-year success rate of the fused root group (75%) was close to that of the separate roots group (76.9%), and the log-rank test showed no statistical significance (*p* = 0.627), as shown in Fig. 4. Finally, the overall success probability after a mean follow-up of 29.74 months was 70.4% (19 of 27 transplants). The unsuccessful tooth was not extracted because the clinical symptoms were not obvious, RCT and periodontal treatment had been undertaken, and follow-up was continuing.

We also built a Cox proportional hazards model, which showed that none of the potential factors [sex, age, recipient site, temporomandibular joint disorder (TMD), simplified oral hygiene index (OHI-S), bruxism, occluding relation] was significantly associated with the success rate (Table 2).

**Table 2:** Omnibus tests of model coefficients

-2 Log Likelihood	Overall (score)			Change From Previous Step			Change From Previous Block		
	Chi-square	df	Sig.	Chi-square	df	Sig.	Chi-square	df	Sig.
30.856	9.114	7	.245	9.889	7	.195	9.889	7	.195
a. Beginning Block Number 1. Method = Enter									

The table above presents the results of the omnibus test sample. The original hypothesis was that ‘the partial regression coefficient of all influencing factors is 0’. However, given that the *P* value obtained from the test is 0.245, which is greater than the significance level of 0.05, we accept the original hypothesis. Therefore, we conclude that there is no factor with a partial regression coefficient that is not zero, and further analysis is unnecessary

### Results of the chewing efficiency test

The absorbance values for each group are summarized in Table 3. The results showed that the transplantation side of the 13 patients had an average chewing efficiency of 82.0% of the healthy side.

**Table 3:** Spectrophotometric value and ratio of transplantation side to healthy side

**Number**	**1**	**2**	**3**	**4**	**5**	**6**	7	**8**	**9**	**10**	**11**	**12**	**13**
Transplanted Side(a)	0.216	0.413	0.288	0.499	0.416	0.434	0.231	0.502	0.458	0.554	0.581	0.332	0.594
Healthy Side(b)	0.339	0.394	0.490	0.616	0.631	0.450	0.244	0.597	0.581	0.727	0.648	0.534	0.541
Ratio (a/b) (%)	63.7	104.8	58.8	81.0	65.9	96.4	94.7	84.1	78.8	76.2	89.7	62.2	109.8

Row a/b was designed to explore the percentage of chewing efficiency of the transplantation side that could be restored to that of the healthy side

**Fig. 5:**
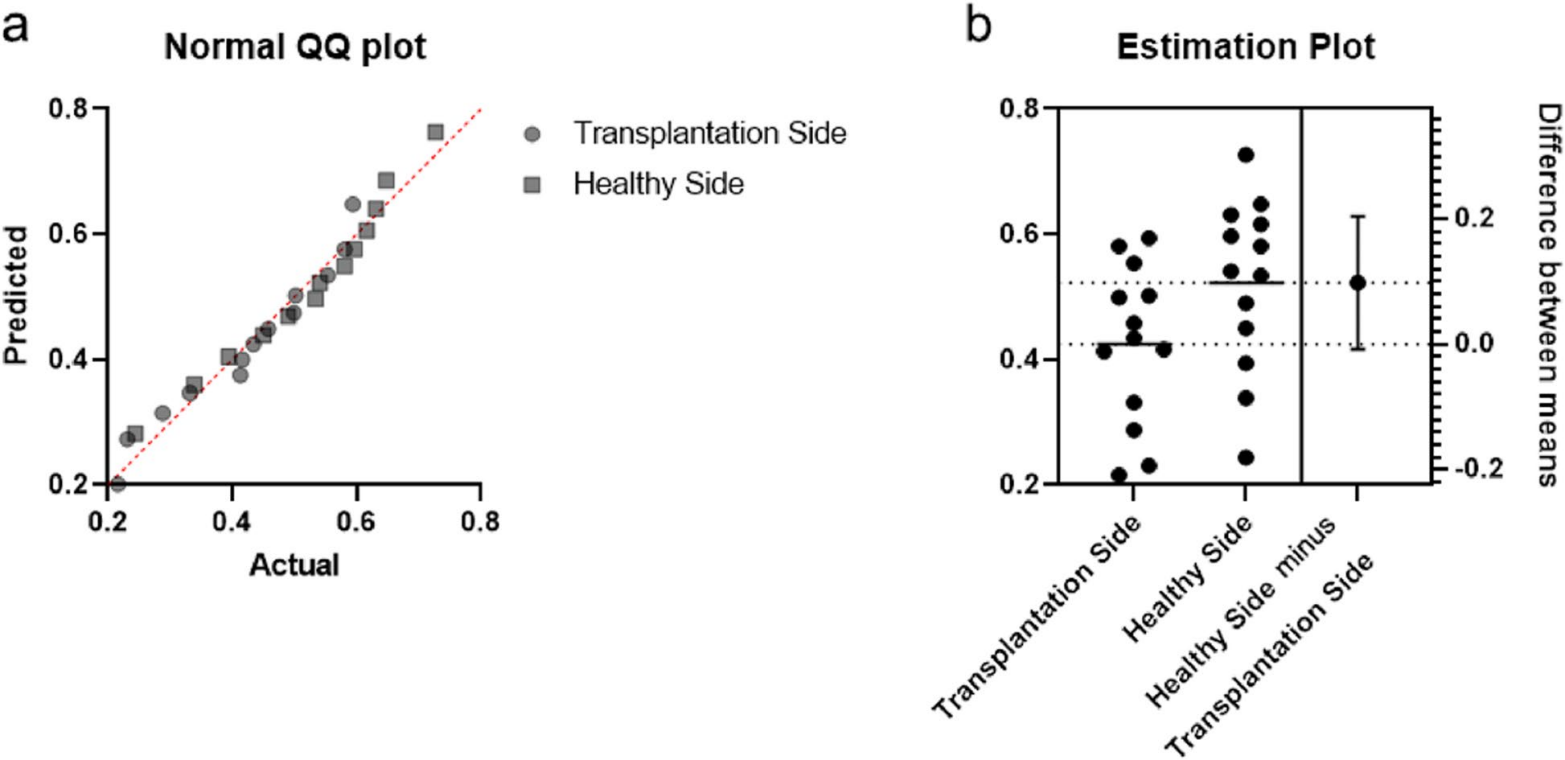
Results of the chewing efficiency test. The results of the chewing efficiency test conform to a normal distribution; A paired t test showed that the differences between the two groups were significant (p < 0.05)

The data from the two groups were normally distributed, and paired t test results indicated significant differences between the two groups (*P < 0.05,* Fig. 5).

### Results of the satisfaction questionnaire

**Fig. 6:**
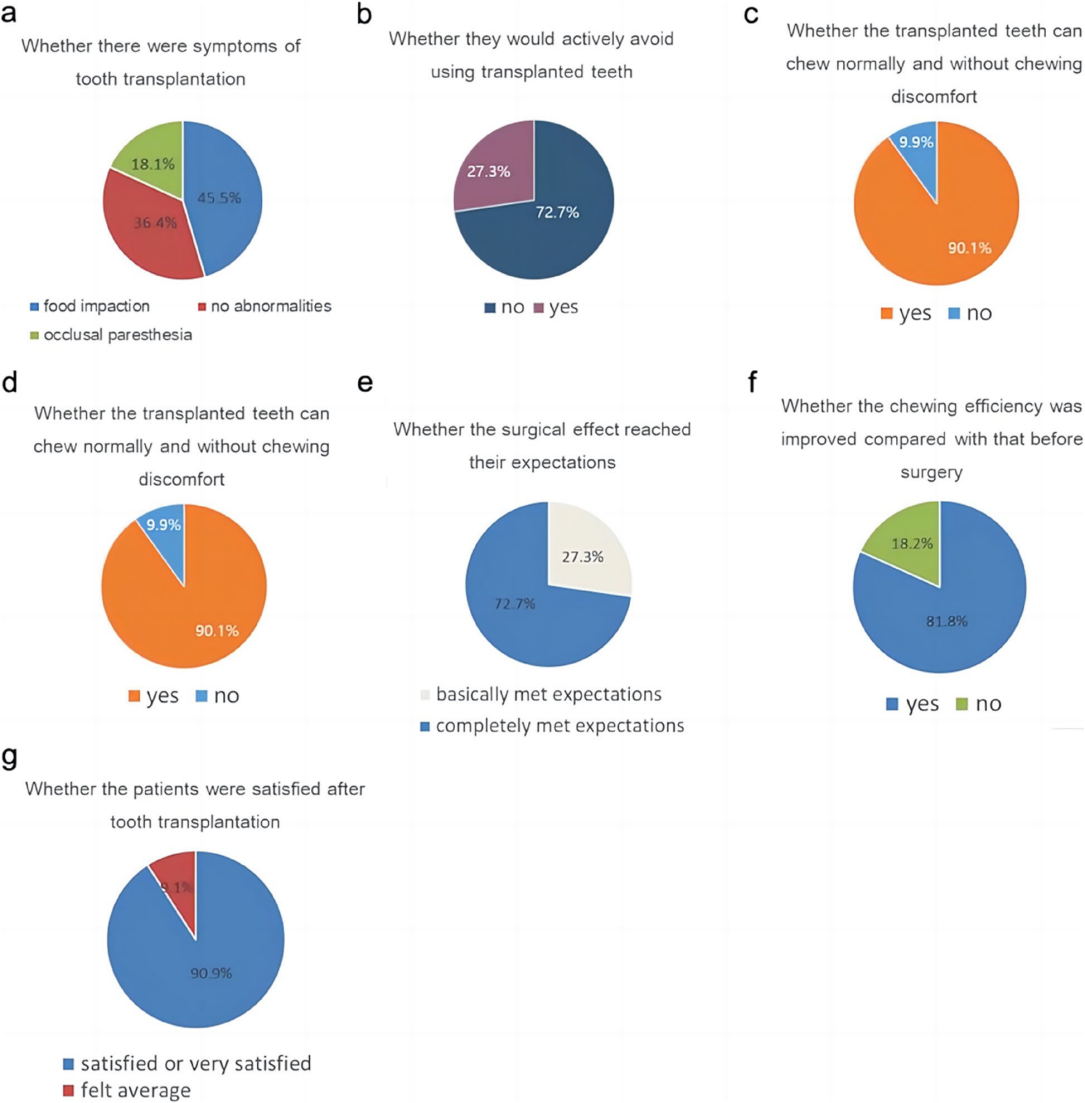
Results of the satisfaction questionnaire

A total of 22 patients participated in the survey and completed the questionnaires. The questionnaire results are presented in Fig. 6.

After tooth transplantation, 45.5% (10) of patients experienced symptoms of food impaction, while 36.4% (8) showed no abnormal symptoms. However, 18.1%(4) of patients reported other symptoms, such as paresthesia during occlusion.

The study found that 72.7% (16) of patients did not actively avoid the use of TT, while 27.3% (6) of patients reported avoidance. Additionally, 90.1%(20) of patients reported being able to chew properly without discomfort while using TT, and 63.6%(14) reported being able to chew hard objects such as nuts and peanuts. However, 27.2% (6) of patients reported being unable to chew hard objects with TT, and 9.1% (2) did not respond to the question.

Of the patients, 81.8%(18) reported a significant improvement in chewing efficiency after surgery, while 18.2%(4) felt that the improvement was not evident.

In terms of whether the surgical results met the patients’ expectations, 72.7%(16) reported that they had fully or largely achieved their desired outcome, while 27.3%(6) stated that the treatment basically met their expectations. Furthermore, in regard to satisfaction with tooth transplantation, 90.9% (20) of patients reported feeling satisfied or highly satisfied, while only 9.1% (2) expressed average or unsatisfactory feelings.

## Discussion

In general, root canal treatment is an essential guarantee for the success of mature permanent tooth autotransplantation. Wisdom teeth, as the most common donor teeth, have complex and variable root canal systems, which increase the difficulty of root canal treatment and thus affect the promotion of autotransplantation, an important technique for natural tooth preservation. This study aimed to explore the possibility of sealing the root canal system without RCT by combining apicoectomy with retrograde filling in ATT. The primary objective was to evaluate the survival rate and success rate of the TT, while secondary objectives included investigating patient satisfaction with the TT, recovery of chewing efficiency and factors affecting the success rate, such as fused root and separate roots, as well as recording relevant clinical and radiographic parameters. The study found that the survival rate of the 27 TTs was 100%, and the success rate was 70.4%. However, the study has certain limitations, including a limited sample size and insufficient follow-up time. More clinical cases and longer follow-up times are required to validate the treatment plan. To the best of our knowledge, this is the first retrospective study to combine extraoral apicoectomy and retrograde filling without RCT specifically on mature molars.

A retrospective analysis was conducted to evaluate the efficacy of extraoral apicoectomy for revascularization of TTs. The study included 35 patients with a mean age of 13 years (range: 8–28 years). The survival rate was 91.4% after a median follow-up of 3.4 years. Of those surveyed, 62.5% had no potential adverse findings. The failure rate was higher in the molar group (72.7%) than in the premolar group (17.6%) and the canine group (25%) [24]. Skoglund’s animal studies demonstrated that apicoectomy does not increase the probability of revascularization [26]. Additionally, another study found that teeth with apical diameters of 0.5–1.0 mm achieved the highest rate of clinical success in revascularization [25]. Our study is primarily focused on tooth transplantation in the molar region, which presents unique challenges due to the fused root, irregular root canal morphology, and smaller roots than anterior teeth and premolars [27, 28]. As a result, it is difficult to obtain the necessary apical foramen diameter required for the revascularization of a mature tooth transplantation after apicoectomy.

Some studies have shown that fused roots can have a complicated root canal system due to canal merging, which can negatively impact apical foramen sealing [29, 30]. However, the Kaplan-Meier 3-year success rate of the fused root group (75%) was similar to that of the separate roots group (76.9%), and the log-rank test did not show any statistical significance (*p* = 0.627). Therefore, our findings suggest that the shape of the root may not be a significant contributor to the success of surgery. Methylene blue staining combined with preoperative CBCT can be used to effectively distinguish the apical foramen after root resection and achieve complete apical sealing [31, 32].

Successful transplantation of mature permanent teeth requires a perfect RCT, which is both sufficient and necessary [6, 8]. For donor teeth with sufficiently developed roots, RCT is commonly initiated within 1–2 weeks postoperation to prevent inflammatory absorption of the root caused by pulp necrosis and to protect the PDL effectively [18, 33]. The primary source of teeth used for ATT are wisdom teeth, which may have varying root canal systems. This can pose a challenge for general dentists when filling the canal, which can limit the adoption of ATT technology. As a result, intraoperative apicoectomy and iRoot BP Plus retrograde filling may be simple and effective methods that can be used to seal the root canal.

Prognostic factors for successful tooth transplantation also involve various factors, such as root development, root morphology, alveolar fossa preparation, and extraoral operation time [1, 13]. However, the patient’s oral hygiene condition is often overlooked. The study found that 5 patients who experienced negative clinical and radiographic outcomes had an OHI-S value of 6. This accounted for 35.7% of all patients with an OHI-S value of 6 or higher. Research studies have consistently demonstrated that practicing proper oral hygiene is crucial in preventing oral inflammation and promoting the regeneration of periodontal tissue [34–36]. Although in the Cox proportional hazards model, none of the potential factors was significantly associated with the success rate (Table 2), we still need to pay enough attention to the oral hygiene status of postoperative patients.

Two patients with bruxism were included in the cases we collected, with one of them having adverse clinical and radiographic findings. Traditionally, bruxism has been considered a contributing factor to periodontal disease, which can affect postoperative healing. However, recent studies have shown an inverse correlation between periodontal disease and bruxism [37]. Bruxers have been found to exhibit better periodontal clinical characteristics and lower odds of developing periodontitis [37–39].

IRoot BP Plus is a bioactive ceramic material that is primarily composed of calcium silicate, calcium carbonate, zirconia, and tantalum oxide. This paste is nontoxic to both pulp and periodontal tissue, which makes it highly biocompatible. It also induces osteogenesis, promotes the regeneration of periodontal tissue, and has excellent sealing, antibacterial properties, and adhesion abilities. These qualities make it a valuable material for use in dental procedures [40, 41]. Compared to MTA, this material can be used in the front teeth without altering their color. Additionally, it does not require any additional preparation before use, which can reduce the time needed for in vitro tooth transplantation [42].

Approximately 10% of third molars with multiple roots have auxiliary root canals that may communicate with periodontal tissue, which could potentially affect our surgical approach [11]. However, we have not observed any negative outcomes resulting from this in our clinical and radiographic examinations, but we still recommend regular postoperative follow-up.

The success of ATT heavily relies on the protection of PDL cells. This can be achieved through aseptic operation techniques, gentle extraction methods, limited exposure time in vitro, and avoiding pressure in the recipient area [7, 43, 44]. These methods are crucial in ensuring the preservation of PDL cells.

Necrotic pulp may cause endodontic absorption after apicoectomy and retrograde filling, which is not present in our case to date. The American Society of Endodontics defines absorption as a physiological or pathological process that results in the loss of dentin, cementum, or bone tissue [45, 46]. Pathological absorption in teeth points to normal pulp tissue granulation denaturation of the pulp tissue, which causes absorption of hard tissue in the tooth body and leads to progressive pathological changes in the dentin layer. It is important to note that the energy source required for cell activity is dependent on the supply from the surrounding tissues. Therefore, as long as there is no accessory canal, the root canal will not be connected to the periodontal tissue, and the occurrence of infection can theoretically be avoided. However, research has shown that necrotic pulp byproducts can infect periapical tissue by travelling through dentinal tubules, which can impact PDL healing [47].

Out of the 27 patients observed, 19 of them had intact crowns, while the remaining 8 had received RCT due to intra- or peri-apical inflammation, but no crown repair was performed. There were no cases of tooth fracture or hidden cracks in any of the patients. Furthermore, the physical and mechanical properties of dentine were not affected by dehydration or RCT [48]. The incidence of tooth fracture following RCT has increased due to the loss of dental structural integrity caused by pathway preparation. Restorations that enhance structural integrity would improve the outcome of RCTs [48].

Tooth pulp necrosis results in crown discolouration, and the main cause of crown discolouration is tooth trauma and bacterial infection caused by pulpitis [49]. Molars and premolars are not located in the aesthetic zone; therefore, crown discolouration has a limited effect on the patient’s appearance. However, the front teeth are in the aesthetic area, and any discolouration can significantly affect the patient’s life. In such cases, the patient may consider veneer repair or bleaching to meet their aesthetic needs. Following apicoectomy and retro-filling, aseptic necrosis developed in the dental pulp within the pulp cavity over time. It becomes uncertain whether the use of calcium silicate bone cement can cause tooth discoloration when blood supply lost.

The results from the chewing efficiency test and questionnaire survey indicate that the majority of patients (90%) reported being able to chew normally with their TTs and can even chew hard objects such as nuts and peanuts (63.6%). Additionally, 72.7% of patients reported not actively avoiding the use of TT. The chewing efficiency of TT is lower than that of implant-supported prostheses due to a mismatch between the crown shape and height of TT and the original molar in the recipient region. This mismatch leads to a reduced contact area between TT and its counterpart [50]. However, the study still revealed a significant improvement in chewing efficiency, with patients achieving 82.0% recovery in comparison to the healthy side (refer to Table 3 for further details). In cases of food impaction, patients can be taught to use dental floss as a conservative approach or ask for a crown restoration to fix the problem.

One of the drawbacks of hydraulic calcium silicate cement is its lower shear bond value, which can have a negative impact on the success rate of this procedure. Xavier et al. conducted a study to investigate the effect of an additional hydrophobic bonding layer(HBL) and repair time on the bonding properties and metamorphic interface between composite cement restorations and calcium silicate-based cement [51]. The results indicated that the application of an additional hydrophobic bonding layer and the timing of restoration have an influence on the bonding properties of the interface between composite adhesive restorations and calcium silicate-based cement. Research has shown that Biodentine has significantly higher microhardness and elastic modulus values compared to other materials, making it a suitable bioceramic cement for use in areas with acidic pH values [52]. Enhancing the adhesion of calcium silicate-based cement may have the chance to improve the sealing effect of IRoot BP Plus on the apical foramen, thereby increasing the success rate of the ATT surgery.

## Conclusion

Extraoral apicoectomy combined with retrograde filling of the TT yielded relatively good results. However, the current study has some shortcomings, such as a small sample size and insufficient follow-up time. In some special cases, such as the complex root canal system of donor teeth that do not warrant perfect RCT, extraoral apicoectomy and retrograde filling to seal the root canal system can be used as alternatives or temporary treatment strategies for TT patients.

## Data Availability

The datasets used and analyzed during the current study are available from the corresponding author on reasonable request.
